# Myopia progression in children during home confinement in the COVID-19 pandemic: A systematic review and meta-analysis

**DOI:** 10.1016/j.optom.2023.100493

**Published:** 2023-10-23

**Authors:** Daisy Laan, Emily T.C. Tan, Paulien I. Huis in het Veld, Hinke Marijke Jellema, Kevin Jenniskens

**Affiliations:** a Clinical Sciences for Health Professionals, Program in Clinical Health Sciences, University Medical Center Utrecht, Utrecht University, The Netherlands; b Department of Ophthalmology, Flevoziekenhuis, Almere, The Netherlands; c Department of Ophthalmology, University Medical Center Amsterdam, Amsterdam, The Netherlands; d Department of Epidemiology, Julius Center for Health Sciences and Primary Care, UMC Utrecht, Utrecht University, Utrecht, The Netherlands

**Keywords:** Myopia, Covid-19, Spherical equivalent, Axial length, Home confinement

## Abstract

**Purpose:**

Myopia is a growing pandemic, especially in children, who risk low vision later in life. Home confinement during the COVID-19 pandemic may have increased myopia progression through increased screentime, decreased time outdoors and increased near work activities. The aim of this study is to compare progression of myopia in children during home confinement period in the COVID-19 pandemic with pre-COVID-19 progression.

**Methods:**

On January 2023 PubMed, EMBASE and Cochrane were searched for relevant studies. Studies meeting the following criteria were eligible for inclusion: children (under 18 years), home confinement due to COVID-19, spherical equivalent refractive (SER) and axial length (AL) measurements and a follow-up period to measure progression. Quality appraisal was performed by two reviewers independently using the Joanna Briggs Institute tool for cohort studies. Outcomes for myopia were assessed through meta-analysis, analyzing SER (random effects) and AL (fixed effects).

**Results:**

Hundred and two articles were identified in the search, of which five studies were included in the analysis. Risk of bias is moderate with a few critical flaws in the studies. Myopia progressed more rapidly during the COVID-19 pandemic compared to the pre-COVID-19 period, both in terms of SER (-0.83D [95 %CI, −1.22, −0.43] and AL (0.36 mm [95 %CI, 0.13, 0.39]).

**Conclusion:**

Progression of myopia during the COVID-19 pandemic accelerated more rapidly compared to the pre-COVID-19 period. Impact of home confinement on myopia may be considered when future lockdown measures are being contemplated.

## Introduction

Myopia (also known as near sightedness), is the fastest growing eye disorder and has been suggested to become an optical pandemic.^1^^,^^2^ Myopia is characterised by an elongated shape of the eye and starts at an early age. The progression of myopia can result in bilateral low vision later in life which substantially compromises quality of life.^2^^,^^3^ The increase in myopia is a global concern. Myopia in Europeans in their twenties has increased from 10 % in the early 70`s, to approximately 50 % in 2020.^2^^,^^4^, ^5^, ^6^, ^7^ In the same period myopia prevalence increased from half of the university students in East Asia to up to 80 % in 2020. ^2^ This leads to a social and economic burden that is expected to further increase in the upcoming decennia.^4^

Lifestyle changes (e.g. excessive use of electronic devices^4^) and external factors (e.g. high pressure education systems^8^) result in decreased time outdoors and increased near work activities.^2^^,^^9^ Prolonged near work activities and indoor location are associated with myopia progression.^4^ When being indoor, objects are typically nearer. This results in a greater hypermetropic peripheral retinal defocus which is a potential driver of elongation of the eye.^10^^,^^11^ Another potential driver of elongation of the eye is the lack of outdoor lighting. Several mechanisms have been suggested to explain this relationship, including the beneficial effects of higher light intensity^12^^,^^13^ (which is 10–1000 times greater than indoor lighting), the potential role of violet light,^14^ and the regulation of the circadian rhythm.^15^^,^^16^ These mechanisms are believed to have a beneficial effect on development of the eye and reduce myopia progression.^12^^,^^13^

In March 2020 the World Health Organization (WHO) declared COVID-19 as a global pandemic.^17^ To contain and mitigate the spread of the pandemic, strict policies came into effect. Public health measures included quarantine, school closure and/or teaching from home, and self-isolation after a positive test result. These measures resulted in home confinement, meaning that an individual needed to stay at home, limiting outdoor activities.^18^ Restrictions were different across countries but a drop in outdoor mobility was seen globally.^19^ Online learning resulted in longer screen time in the daily life of children.^20^

Although studies on the effects of home confinement during COVID-19 have been performed, an overview of literature is still lacking.

The aim of this study is to evaluate if progression of myopia in children increased during home confinement periods in the COVID-19 pandemic compared to the pre-COVID-19 era.

## Methods

A systematic review was conducted following the Cochrane guidelines.^21^ Guidelines for reporting this study are in accordance with the Preferred Reporting Items for Systematic Reviews and Meta-Analysis (PRISMA) statement.^22^ A search of the literature was conducted in the electronic databases PubMed (National Library of Medicine), EMBASE and Cochrane Library. Citation searching was performed through Scopus.

The search query included terms related to “myopia”, “children”, “quarantine”, “COVID-19″, “education”, “screen time”, “ocular refraction” and “axial length”. The complete search is available in Appendix 1.

All studies published between March 1, 2020, and January 4, 2023, identified in the search were included for title and abstract screening. Full text published studies were included when the following criteria were met: children under the age of 18 years old, spherical equivalent refraction (SER), axial length (AL) and a follow-up period to measure progression. Date restriction was applied to ensure only studies performed during or after the COVID-19 pandemic were included. Studies were excluded if the article was not available in English, a commentary, perspective, a letter to the editor or survey. Studies were also excluded when patients had prior myopia management therapy like optical intervention with contact lenses, defocus incorporated multiple segment (D.I.M.S) spectacle lenses or pharmaceutical intervention with atropine.

All literature searches were imported in the software program Mendeley Reference Manager.^23^ After removing duplicates, two reviewers (D.L. and E.T.) assessed the studies independently for eligibility based on title and abstract. Secondly full-text studies were sought for retrieval and assessed for eligibility.

The extracted data from the included articles were obtained by one researcher (D.L). The demographic table included: authors, publication year, sample size, geographic location, the mean age of study participants including the standard deviation, gender, follow-up period and myopia prevalence. The extracted data also included the main outcomes: SER (in diopter) and AL (in mm) baseline values including the change over the follow-up period. To ensure comparability of studies with varying follow-up periods, a normalized follow-up period of 12 moths will be calculated using linear extra- or interpolation. The formula used for this linear extrapolation for Normalized Effect = (12 x Study Effect) / (Study Follow-Up).^24^ The distribution of AL among the participants within each study will be plotted relative to age, generating a growth chart. Growth charts are used to detect abnormal growth in a child. Depending on the child's ethnicity, different growth curves are available. The growth chart of Tideman et al.^25^ is developed for European children and He et al.^26^ is developed for Asian children. In this study the choice for He et al.^26^ is made due to the similar geographical location of the execution of the included studies.

Additionally, there is assessed whether the included studies in our analysis examined the impact of other lifestyle factors such as outdoor time and near work activities.The methodological quality of the included studies was independently assessed by two reviewers (D.L. and E.T.). Disagreements were resolved between authors through discussion.

The selected studies were critically appraised using the Joanna Briggs Institute (JBI) checklist for cohort studies.^27^

A meta-analysis approach was used for the synthesis of the data to compare the study results. Analysis and formulas were entered in Microsoft Excel 2010 (Microsoft, Redmond, WA, USA). The data analysis was checked using Review Manager (version 5.4.1; Cochrane Collaboration).^28^ Changes in SER and AL were analyzed and the standardized effect sizes were calculated using Hedges` g. Forest plots were created and a pooled estimate was calculated using the inverse variance weighting method. Statistical heterogeneity was assessed using I^2^. Depending the outcome of the I^2^ test and visual inspection, the decision was made to execute a fixed effects model (I^2^<50 %) or a random effects model (I^2^>50 %).

## Results

The primary search resulted in 182 records. After duplicate removal, 102 records were screened on title and abstract. A total of 49 studies were included in full-text screening.

**Fig. 1:**
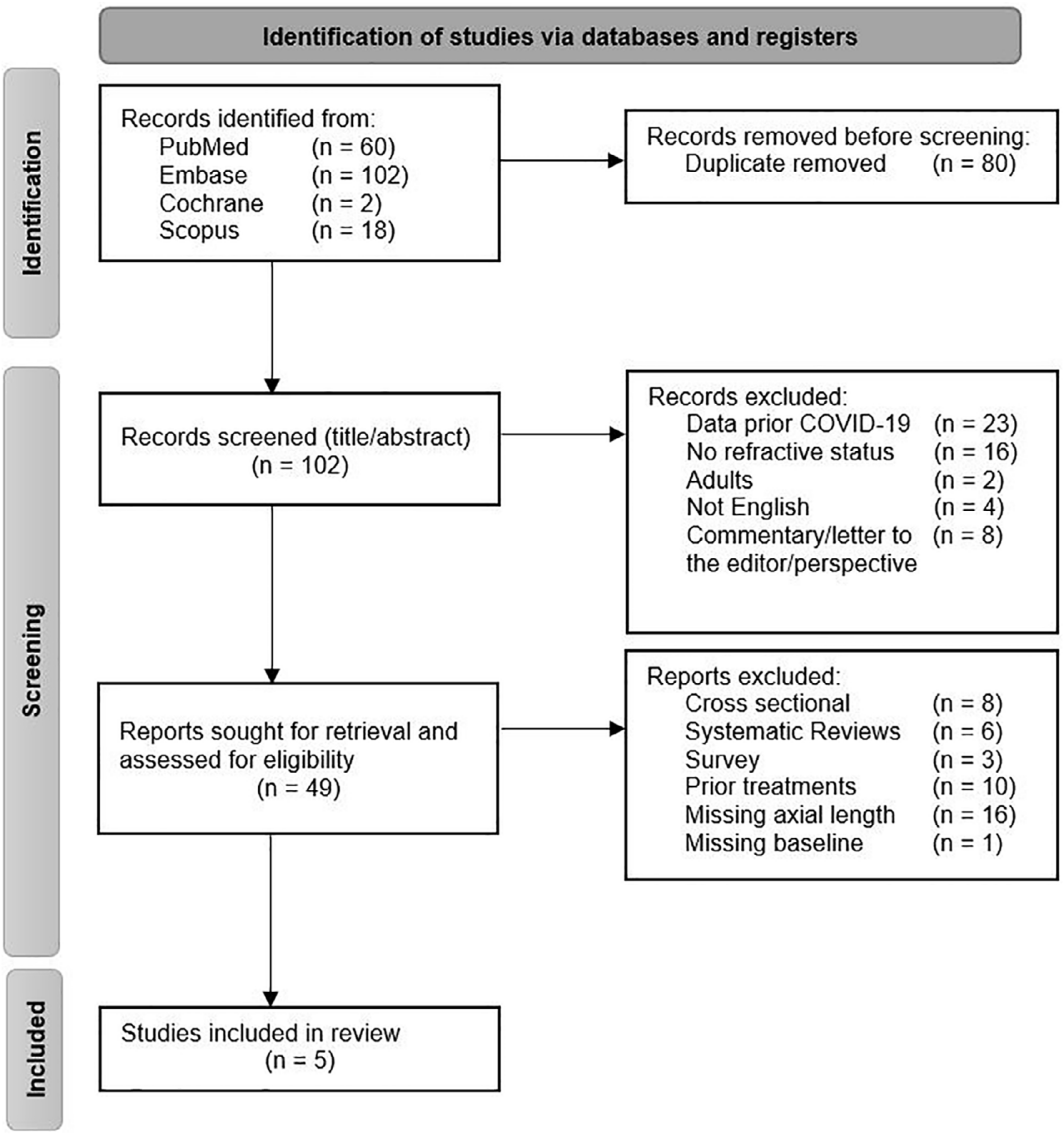
PRISMA Flowchart. Identification of studies via databases are included in the PRISMA Flowchart. Duplicates are removed and excluded reports are sorted by given reason.

After full-text screening 44 studies were excluded for not meeting the inclusion criteria. A total of five cohort studies were included in the review for analysis (Fig. 1).^24^^,^^29^, ^30^, ^31^, ^32^

The demographic results from the five included cohort studies can be found in table 1. All studies were observational cohorts conducted in Asia. A total of 4566 children were included in this meta-analysis of which 2181 children were exposed to COVID-19 restrictions with home confinement. In all studies the control group consisted of a matched selection of children who have the same age and geographic location as the exposure group but received their follow-up period before the COVID-19 pandemic. Follow-up ranged from 7^30^^,^^31^ to 36^32^ months. In two studies the follow-up period differed between the exposed and control groups within those studies^32^.

**Table 1:** Demographic results. Per study the demographic results are displayed. The amount of patients per exposed and control group. The distribution of age at baseline is represented with a mean and standard deviation. The proportion male is shown. Per study the data collection period for the control group is before the COVID-19 period and for the exposure group after regulations of COVID-19 were applied.

Author, Year	Sub group	N	Location	Baseline Age (years) [SD]	Male, %	Data collection period
Choi, 2022^24^	E	56	Hong Kong	10.8 [1.50]	48	June 2019–May 2021
	C*	81		10.0 [1.45]	54	Aug 2014–July 2017
Hu, 2021^29^	E	1054	China	7.76 [0.32]	53	Nov 2019–Nov 2020
	C	1060				Nov 2018–Nov 2019
Ma, 2021^30^	E	208	China	8.90 [0.69]	52	Jan 2020–Aug 2020
	C	83				April 2018–Dec 2018
Ma, 2022^31^	E	77	China	8.65 [0.29]	52	July 2019–Aug 2020
	C	77				Sept 2015–Sept 2016
Zhang, 2021^32^	E	709	Hong Kong	7.25 [0.92]	NA	Dec 2019–Aug 2020
	C	1084		7.29 [0.75]		Jan 2015–Jan 2020

N, amount of patients in study; E, exposure group; C, control group; mo, months D, diopter; mm, millimeter; SER, spherical equivalent refraction; AL, axial length; [SD], standard deviation; NA, not available; *Control group from a study of Lam et al., 2022.^33^

From the study of Choi et al.^24^ the data of the control group with single vision lenses (SVL) were used as exposure group in this systematic review. The other group from this study is excluded due to the intervention with defocus incorporated multiple segments technology (DIMS) which have a therapeutic inhibitory effect on the progression of myopia.^24^ To compare the SVL group, who were exposed to COVID-19 regulation, the authors included a control group^33^ with a comparable study design conducted in the same geographical location prior to the COVID-19 pandemic.

### Methodological quality

Table 2 shows the results of the quality appraisal. Group selection and exposure measures were low risk of bias. All studies had participants with similar characteristics in age, sex and geographical location in the exposure group compared to the control group. All exposed participants endured home confinement during the COVID-19 period and all control groups were included before the COVID-19 period.

**Table 2:** Joanna Briggs Institute (JBI) Critical Appraisal Tool. The methodological quality of included studies is assessed with the JBI critical appraisal checklist for cohort studies. Whenever a component is applicable it was scored with a ‘yes’. When the component does not apply for the study, it was scored with ‘not applicable’. If the study lacks the component, the score ‘no’ was applied. Whenever it was uncertain if a component is applicable, it was scored with ‘unclear’.

Author, Year	1. Similar Groups	2. Exposure measure EG/CG	3. Exposure measure valid	4. Confounders identified	5. Confounder strategies	6. Start free of outcome	7. Outcome measure valid	8. Follow-up time	9. Follow-up complete	10. Follow-up strategies	11. Statistical analysis appropiate
Choi, 2022^24^	**YES**	**YES**	**YES**	**YES**	**YES**	NO	**YES**	**YES**	UN	UN	**YES**
Hu, 2021^29^	**YES**	**YES**	**YES**	NO	NA	NO	**YES**	**YES**	NO	NO	**YES**
Ma, 2021^30^	**YES**	**YES**	**YES**	**YES**	**YES**	NO	**YES**	UN	UN	UN	**YES**
Ma, 2022^31^	**YES**	**YES**	**YES**	NO	**YES**	NO	**YES**	UN	UN	UN	**YES**
Zhang, 2021^32^	**YES**	**YES**	**YES**	**YES**	**YES**	NO	**YES**	UN	UN	UN	**YES**

UN, unclear; NA, not applicable; EG, exposure group; CG, control group.

All studies were observational and included appropriate statistical analysis. Three studies were at higher risk of bias due to a limited follow-up period of less than one year. It is unclear if this follow-up period is sufficient for effects of myopia due to homeconfinement to manifest.^30^, ^31^, ^32^ None of the studies used statistical methods or additional analyses to address loss to follow-up. Identification of confounders varied in the included studies. An attempt was made to consider confounding by including the degree of lockdown.^24^ Furthermore questionnaires were completed to investigate the risk for myopia.^30^, ^31^, ^32^

### Spheric equivalent refraction

The baseline of SER of the included participants in the studies differed (table 3). One study only included myopic children.^24^ In the four other studies all refraction types were included.^29^, ^30^, ^31^, ^32^ In three of these studies the different refractions were categorized.^29^, ^30^, ^31^ Ma et al.^30^ categorized 90 myopes, 77 emmetropes and 41 hyperopes out of the 208 included patients the exposure group. Among the 83 children in de control group, there were 83 myopes, 25 emmetropes and 20 hyperopes. Hu et al.^29^ had a larger sample size and consisted of 85 myopes, 1041 emmetropes and 81 hyperopes out of the 1207 included patients in the exposure group. Among the 1472 children in the control group there were 115 myopes, 1262 emmetropes and 95 hyperopes. The third study that included all refractions, Ma et al.,^31^ only showed the amount of myopes in the groups. The two similar groups of 77 patients both included 25 myopic children.

**Table 3:** Results of main outcome measurements. Information throughout the study from mean baseline SER and AL until the last follow-up is displayed. Every study had its individual follow-up time. The normalized difference between baseline and follow-up is based on a follow-up time of 12 months.

Author, Year	Group	N	Follow-up, Months	Spheric equivalent refraction, D [SD]				Axial length, mm [SD]			
				Baseline	Follow-up	∆	Norm ∆	Baseline	Follow-up	∆	Norm ∆
Choi, 2022^24^	E	56	14	−2.99 [0.26]	NA		−0.56 [0.46]	24.84 [0.18]	NA		0.29 [0.18]
	C*	81	24	−2.76 [0.96]		−0.85 [0.72]	−0.43 [0.72]	24.60 [0.83]		0.55 [0.18]	0.28 [0.18]
Hu, 2021^29^	E	1054	12	0.86 [0.94]	0.20 [1.15]	−0.67 [0.56]	−0.67 [0.56]	22.92 [0.74]	23.23 [0.79]	0.31 [0.24]	0.31 [0.24]
	C	1060		0.82 [1.06]	0.55 [1.16]	−0.31 [0.46]	−0.31 [0.46]	23.03 [0.75]	23.25 [0.78]	0.22 [0.21]	0.22 [0.21]
Ma, 2021^30^	E	208	7	−0.50 [1.25]	NA	−0.93 [0.65]	−1.59 [0.65]	23.08 [0.92]	NA	0.24 [0.19]	0.41 [0.19]
	C	83		−0.47 [1.38]		−0.33 [0.47]	−0.57 [0.47]	23.85 [0.94]		0.21 [0.39]^b^	0.36 [0.39]^b^
Ma, 2022^31^	E	77	7	−0.26 [0.93]	NA	−0.83 [0.56]	−1.43 [0.56]	23.18 [0.72]	NA	0.20 [0.20]^a^	0.34 [0.20]^a^
	C	77		−0.14 [1.09]		−0.28 [0.54]	−0.72[0.54]	23.21 [0.71]		0.20 [0.12]^a^	0.34 [0.12]^a^
Zhang, 2021^32^	E	709	8	0.32 [1.16]	−0.19 [1.33]	−0.50 [0.51]	−0.75 [0.51]	22.98 [0.83]	23.27 [0.87]	0.29 [0.35]	0.44 [0.35]
	C	1084	36	0.34 [1.49]	−0.93 [2.14]	−1.27 [1.34]	−0.42 [1.34]	23.02 [0.91]	23.89 [1.11]	0.88 [0.49]	0.29 [0.49]

E, exposure group during COVID-19; C, control group before COVID-19; D, dioptre, mm, millimeter; [SD], standard deviation; NA, not applicable; ∆, difference between follow-up and baseline; Norm ∆, normalized for 12 months follow-up with: difference between baseline and follow up of SER or AL × (12/months between baseline and follow-up); *, Control group from a study of Lam et al., 2022.^33^

a Standard Deviation calculated from Interquartile range.

b Standard Deviation collected from figure.

At the follow up endpoint myopia prevalence, reported in two studies, was higher in the exposure group than the control group.^29^^,^^32^ Hu et al. measured a 7.5 % higher myopia prevalence in the exposure group after 12 months.^29^ The study of Zhang et al.^32^ had a higher prevalence rate in the control group. However, their control group data collection period is three years compared to eight months in the exposure group. Calculating the normalized myopia progression of one year results in a higher myopia prevalence of 13.1 % in the exposure group (24.42 %) compared with the control group (11.32 %).

Hu et al.^29^ not only categorized the participants on different refraction types, as mentioned before, but also used this in their analysis. In the exposure and control group, the major part was emmetropic (86.3 % versus 85.7 %). Myopia (7.0 % versus 7.8 %) and hyperopia (6.7 % versus 6.5 %) were less frequently present in the included baseline. After the follow-up period participants with myopia at both visits had the largest myopia progression. Participants with hyperopia at both visits had the smallest myopia progression during the follow-up period.

All studies show more progression in mean SER in the exposure group compared to the control group over a calculated 12 month period perspective. This effect size of progression of SER between exposure and control group differs per study. Choi et al.^24^ reports the smallest effect size (−0.21 D) and Ma et al.^30^ reports the largest effect size (−1.68 D).

**Fig. 2:**
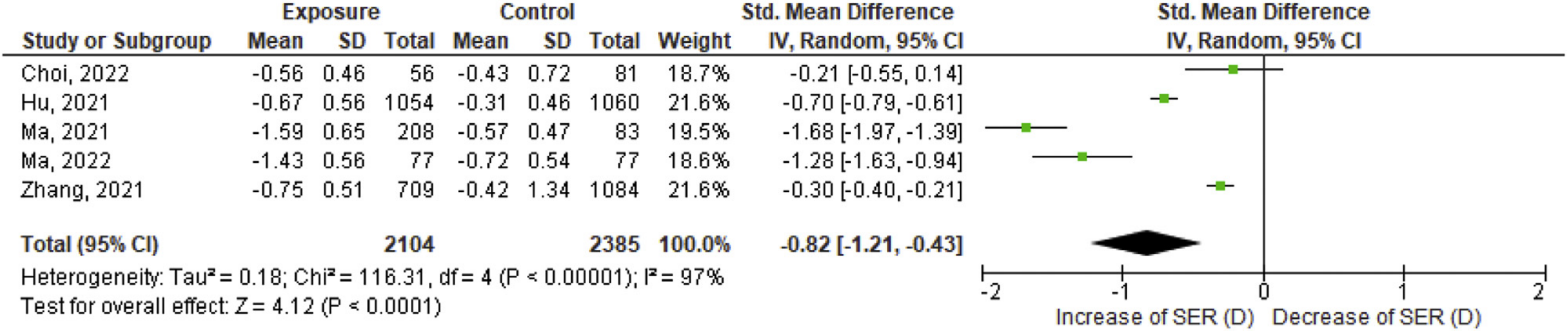
Effect size of spherical equivalent refraction (SER) in diopter. For each study, SER data from exposed and control group are retrieved in effect size data including standard deviation. All included studies together give the pooled effect. The bold line at ‘0′ represents no difference in effect size of SER between the two groups. The left side of the chart implies an increase in SER towards myopia.

These results including the corresponding 95 % confidence interval (CI) are graphically shown in Fig. 2. The statistical test I^2^ for heterogeneity is 97 %, therefore a random effects model was executed. The pooled effect of the five studies is −0.82 D [95 % CI, −1.21, −0.43].

### Axial length

The baseline differences in mean AL between the exposed and control groups were small in three studies (0.03^31^, 0.04^32^ and 0.11 mm^29^) in which stratified cluster sampling is applied. The two other studies (0.24^24^ and 0.77 mm^30^) showed a substantial difference in the mean baseline values of AL.

**Fig. 3:**
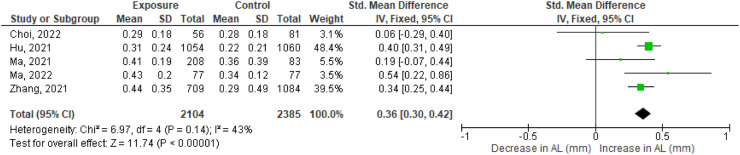
Effect size of axial length (AL) in mm. For each study, AL data from exposed and control group are retrieved in effect size data including standard deviation. All included studies together give the pooled effect. The bold line at ‘0′ represents no difference in effect size of AL between the two groups. The right side of the chart implies a growth of AL.

Normalized progression of AL calculated for 12 months was longer in the exposure group than the control group in three out of the five studies.^29^^,^^31^^,^^32^ This difference in AL between the exposed and control group was 0.19^30^, 0.34^32^ and 0.40^29^ mm. Another study showed a small difference of 0.06^24^ mm, the fifth study showed no change in AL progression between the exposure and control group.^31^ These results, including the corresponding 95 % confidence interval (CI), are shown in Fig. 3. The statistical test I^2^ for heterogeneity is 43 %, therefore a fixed effects model was performed. In the forest plot, three studies had a CI crossing zero meaning there was no total effect. The pooled effect size is 0.36 mm [95 % CI, 0.30, 0.42].

**Fig. 4:**
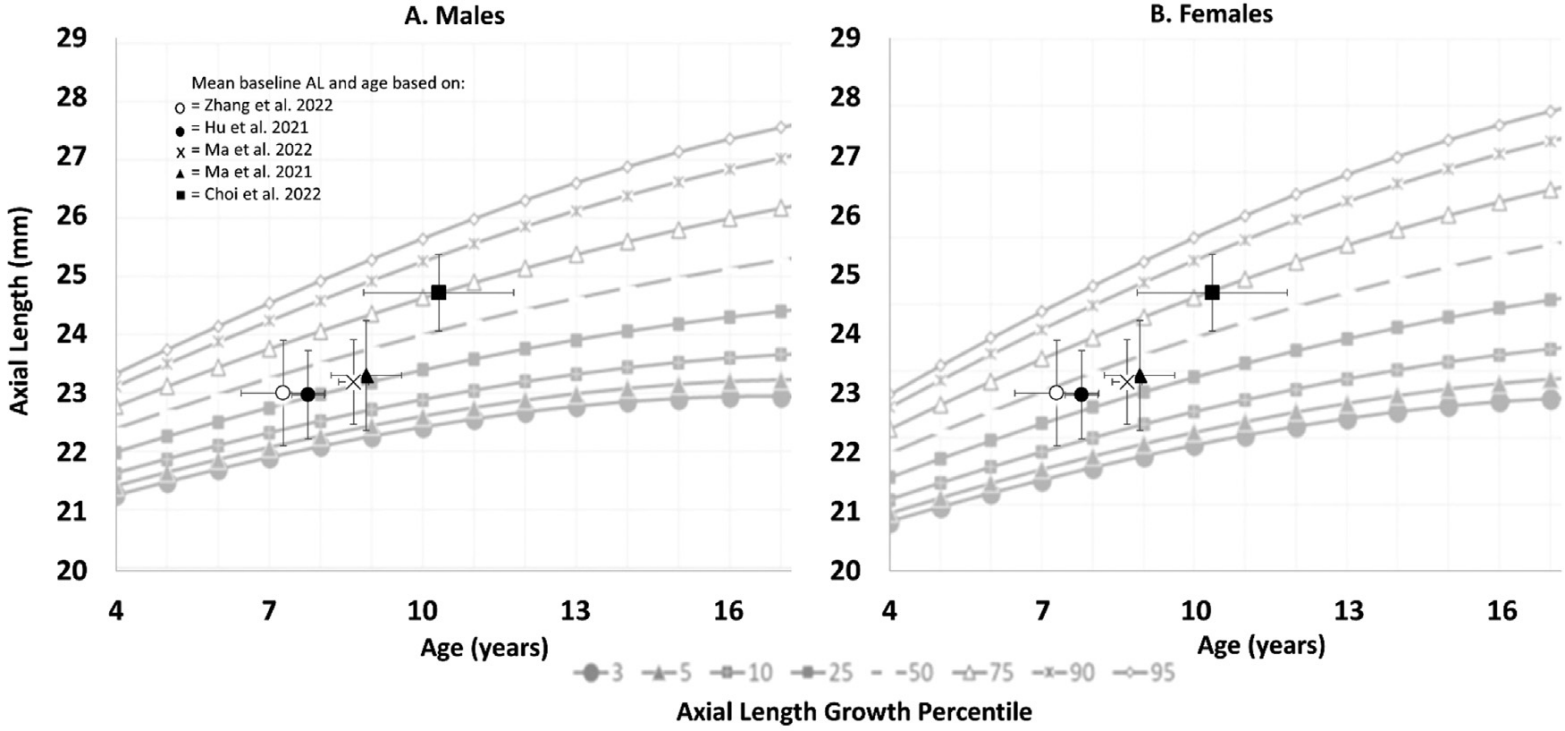
Axial length (AL) growth percentile. For this figure the growth percentile chart from He et al. 2023^26^ is used with in (A) the male growth curve and (B) the female growth curve. The *y*-axis represents the distribution of the AL in mm. The *x*-axis represents the distribution of age in years. Underneath the figure the distribution of axial length growth percentile is illustrated. Each included study is plotted in the chart with error bars based on the overall mean baseline values of the whole study population. The horizontal error bar includes the standard deviation of age, the vertical error bar includes the standard deviation of AL.

Plotting the AL baseline measurements of the included studies on the AL growth chart shows an unequal allocation (Fig. 4). The gender distribution in the included studies was rather equal, therefore both gender growth charts were used. Four studies are ranked in the 25th percentile^29^, ^30^, ^31^, ^32^ and one study is ranked in the 75th percentile.^24^

### Outdoor and near work time

In three studies the participants completed a questionnaire to investigate the risk factors for myopia^30^, ^31^, ^32^ The questionnaire focused on near work and outdoor activity-related parameters.

The outdoor time decreased with 0.86^31^, 0.85^30^, and 0.8632 h/day in the COVID-19 period. The near work time increased with 3.12^31^, 3.33^30^, and 4.5832 h/day. Zhang et al.^32^ calculated that the outdoor time decreased approximately 3.1 fold and the near work time increased approximately 2.8 fold.

## Discussion

This systematic review and meta-analysis reports the refractive change in children due to home confinement in the COVID-19 pandemic. Progression of myopia accelerated more rapidly in this period compared to control groups pre-COVID-19. The SER increased (−0.82D [95 %CI, −1.21, −0.43]) and the AL (0.36 mm [95 %CI, 0.30, 0.39]) elongated even more than before the COVID-19 pandemic.^24^^,^^29^, ^30^, ^31^, ^32^,^30^, ^31^, ^32^

All studies included in the analysis were observational of nature and implemented appropriate statistical methodologies. The critical appraisal assessment revealed overall good quality, with the section on follow-up time showing weaknesses. Specifically the duration of the follow-up period which was less than a year in three studies,^30^, ^31^, ^32^ raising questions about its adequacy. None of the studies used a strategy for loss of follow-up.

Results from studies with follow-up that were included in this paper are in line with other studies using different designs. Comparing this longitudinal meta-analysis to cross-sectional studies the higher prevalence of myopia due to home confinement is confirmed.^34^, ^35^, ^36^, ^37^ It is also known that fewer outdoor activities and more near work lead to an increase in myopia.^11^^,^^38^, ^39^, ^40^ During the quarantine there is more time spend indoor and more time spend on near work activities.^41^^,^^42^ The lifestyle questionnaires in the included studies also showed a decrease in outdoor activity and an increase in near work activity.^30^, ^31^, ^32^ During the home confinement period the eye elongated more than usual for the age. The regular growth of the eye can be seen with the growth charts for Asian children.^26^ The AL of the included studies from this meta-analysis are plotted on and below the median line of the growth chart for Asian children.^26^ Hence, it is reasonable to suggest that with the exception of one study,^24^ these participants can be categorized as non-fast progressors. Nevertheless, the overall effect size shows an elongation of an additional 0.36 mm on top of the regular yearly progression in this study.

The progression of myopia during the COVID-19 period can be related to the applied restrictions like home confinement. Children spent less time outside and focussed for prolonged hours at near vision distance. However, the exact cause is not fully understood, a recent study^43^ found that during the COVID-19 period, myopia progression was associated with time spent on digital screens rather than outdoor activities. This gives us an insight into the potential impact of current lifestyle changes, such as increase in technology use, gaming and the high-pressure education systems.^2^^,^^4^^,^^9^ Change in lifestyle present due to the COVID-19 restrictions has a major impact on the health of the eyes of children and for them later in life. More children develop myopia and are at risk of obtaining high myopia with the consequences to acquire low vision later in life. If a future pandemic would occur, resulting in home confinement, prevalence of myopia may increase even further. This could lead to higher economic costs as well as a social burden to quality of life.^4^

There is a difference in progression among the different refractive categories. Participants with hyperopia at both visits had the smallest progression during the follow up period.^29^ This is consistent with the known observation that myopic refractions tend to progress faster compared to other refractive errors.^25^ Therefore, baseline refractions should be considered. According to Hu et al.^29^ the participants who were myopic at both visits were most likely to increase in SER and AL. This suggests that this study population is expected to have a faster natural growth rate.

However, in this review, two studies^24^^,^^30^ deviate from the trend observed in AL progression. Choi et al.^24^ showed that inclusion of only myopic participants did not lead to the most progression compared to the other studies. This discrepancy can be influenced by a form of selection bias for two reasons. 1) The SD of age was much higher in the study of Choi et al.^24^ and 2) no lifestyle questionnaire was conducted like the other included studies in this meta-analysis.^30^, ^31^, ^32^ Therefore, they might have had a lack of outdoor exposure, longer near work duration or a closer near work distance before the home confinement.^44^ Ma et al.^30^ is the second study that does not align with the general trend in AL progression. A possible explanation is the baseline AL of the exposure and control groups, with the exposure group classified in the 25th percentile and the control group in the 75th percentile. This would be expected to result in a higher overall progression rate for the control group, but interestingly, the exposure group demonstrates a larger increase in AL. Which still confirms the higher progression rate during the COVID-19 period. A strength of this meta-analysis is that only longitudinal study designs were used. More cross-sectional data is available on myopia progression in the COVID-19 pandemic.^34^, ^35^, ^36^, ^37^ The inclusion of follow-up time allows assessment of actual growth of SER and AL per participant over time. Heterogeneity in follow-up periods was addressed by using a normalized follow-up period, allowing for comparison and pooling between studies.

Some limitations need to be addressed in this meta-analysis as well. Due to the stringent inclusion criteria, we excluded several studies which did not include AL. Another limitation is the applicability of the findings to the non-Asian setting. The prevalence of myopia in Asia of around 80 % is higher compared to 50 % in the young adults in Europe.^2^^,^^6^^,^^7^ Despite genetic predisposition, environmental factors play a role in this difference. High pressure education systems can cause lifestyle differences.^2^ Asian countries are already exposed to a higher level of near work time and less outdoor time since many more years compared to European countries.^4^ Contrarily, low-income countries are more likely to have a shorter duration of near work time before the COVID-19 pandemic which might result in a larger impact after home confinement is applied. Additionally, it is important to acknowledge that the inclusion of publications during the COVID-19 pandemic resulted in a time frame of 2–3 years, which could also potentially affect the implication of the global trend of myopia progression. Holden et al.^2^ had already described the trend of myopia progression prior to the pandemic. It is possible that an increase in myopia could have occurred even without the pandemic, and the extent to which the pre-COVID trend has been further shifted with this study design is unknown. Therefore, the observed progression is likely a combination of the long-term trend and the recent COVID-19 effect.

The evidence for clinical recommendations is based on the available studies. All studies were observational, and the strengths were moderate. Recommendations are therefore of moderately importance to the outcome.

Future studies should use a longer follow up time of at least 12 months to ensure that the effect of home confinement is exerted. Pooled data showed a difference in SER and AL growth between the exposure group and control group, but it would be preferable to have a longer follow-up time. In addition, future research should administer questionnaires on the duration and frequency of outdoor and near work activities, so that more insight on the causal etiologic factors of myopia can be acquired.

In conclusion, myopia in children progressed more rapidly during the COVID-19 pandemic, when home confinement was common, compared to pre-COVID-19 era. Outdoor time decreased and near work time increased due to home confinement during the COVID-19 pandemic.

Home confinement may provide insight in future lifestyle changes when near work increases and outdoor time decreases. Modification of lifestyle changes should be implemented during home schooling and awareness in educational programs for children is strongly advised to prevent further escalation of myopia progression in children.

## Figures and Tables

**Table:** 

Search Strategy		
**#**	**Search**	**Results**
1	“Myopia”[MeSH] OR “Myop*”[tiab] OR “Myopia control”[tiab] OR “Myopia management”[tiab] OR “Myopia progres*”[tiab] OR “High Myopia”[tiab] OR “Progressive Myopia”[tiab] OR “nearsight*”[tiab] OR “Child”[MeSH] OR “Child*”[tiab] OR “Adolescen*”[tiab]	PubMed (n = 165,517) Embase (n = 4,479,571) Cochrane (n = 283,379) Scopus (n = 4,682,353)
2	“Quarantine”[MeSH] OR “Quarant*”[tiab] OR “Lock down”[tiab] OR “Stay at home orders”[tiab] OR “Health lockdown*”[tiab] OR “Self-Quarantine”[tiab] OR “Self Quarantine”[tiab] OR “Cordon Sanit*”[tiab] OR “COVID-19”[Mesh] OR “COVID-19”[tiab] OR “Pandemics”[Mesh] OR “pandemic*”[tiab] OR “online learning”[tiab] OR “SARS-CoV-2”[Mesh] OR “SARS-CoV-2”[tiab] OR “SARS-CoV-2 Virus*”[tiab] OR “SARS-CoV 2 Virus*”[tiab] OR “Virus, SARS-CoV-2”[tiab] OR “SARS Coronavirus 2”[tiab] OR “Coronavirus 2, SARS”[tiab] OR “Coronavirus Disease 2019 Virus”[tiab] OR “2019 Novel Coronavirus*”[tiab] OR “Coronavirus, 2019 Novel”[tiab] OR “Novel Coronavirus, 2019”[tiab] OR “Wuhan Seafood Market Pneumonia Virus”[tiab] OR “2019-nCoV”[tiab] OR “COVID-19 Virus*”[tiab] OR COVID 19 Virus*[tiab] OR “Virus, COVID-19”[tiab] OR “Wuhan Coronavirus*”[tiab] OR “COVID19 Virus*”[tiab] OR “Severe Acute Respiratory Syndrome Coronavirus 2”[tiab] OR “Education, Distance”[Mesh] OR “Education, Distance”[tiab] OR “Distance Education”[tiab] OR “Distance Learning”[tiab] OR “Learning, Distance”[tiab] OR “Online Learning”[tiab] OR “Learning, Online”[tiab] OR “Online Education*”[tiab] OR “Correspondence Cours*”[tiab] OR “Screen Time”[MeSH]	PubMed (n = 370,19) Embase (n = 636,149) Cochrane (n = 11,827) Scopus (n = 218,287)
3	“Refraction, Ocular”[Mesh] OR “Refraction, Ocular”[tiab] OR “Ocular Refract*”[tiab] OR “progression of myopia”[tiab] OR “Myopia progress*”[tiab] OR “Axial Length, Eye”[Mesh] OR “Axial Length, Eye”[tiab] OR “Eye Axial Length*”[tiab]	PubMed (n = 17,921) Embase (n = 20,714) Cochrane (n = 1,109) Scopus (n = 15,489)
4	#1 AND #2 AND #3	PubMed (n = 60) Embase (n = 102) Cochrane (n = 2) Scopus (n = 18)
